# Rainforest and cloud forest *Scolytodes* (Curculionidae, Scolytinae, Hexacolini) from the Arthropods of La Selva inventory in Costa Rica: new species, new synonymy, new records

**DOI:** 10.3897/zookeys.863.33183

**Published:** 2019-07-11

**Authors:** Bjarte H. Jordal, Lawrence R. Kirkendall

**Affiliations:** 1 University Museum of Bergen, PO 7800 N-5007 Bergen, Norway University of Bergen Bergen Norway; 2 Department of Biological Sciences, University of Bergen, PO 7800, N-5020 Bergen, Norway University of Bergen Bergen Norway

**Keywords:** bark beetles, biodiversity survey, taxonomy, transect, weevils

## Abstract

Quantitative collecting efforts over the last several decades in Costa Rica have resulted in many new species of insects. The Arthropods of La Selva projects included collecting from a typical lowland Neotropical forest and up an altitudinal transect, and has provided many valuable samples of insects, spiders and mites potentially new to science. We describe 18 new species in the bark beetle genus *Scolytodes* Ferrari, 1867, 14 of which were collected during this project: *S.angulus* Jordal & Kirkendall, **sp. nov**., *S.sufflatus* Jordal & Kirkendall, **sp. nov**., *S.squamatifrons* Jordal & Kirkendall, **sp. nov.**, *S.comosus* Jordal & Kirkendall, **sp. nov.**, *S.spatulatus* Jordal & Kirkendall, **sp. nov.**, *S.seriatus* Jordal & Kirkendall, **sp. nov.**, *S.profundus* Jordal & Kirkendall, **sp. nov.**, *S.catinus* Jordal & Kirkendall, **sp. nov.**, *S.fimbriatus* Jordal & Kirkendall, **sp. nov.**, *S.sulcifrons* Jordal & Kirkendall, **sp. nov.**, *S.planifrons* Jordal & Kirkendall, **sp. nov.**, *S.porosus* Jordal & Kirkendall, **sp. nov.**, *S.mundus* Jordal & Kirkendall, **sp. nov.**, *S.callosus* Jordal & Kirkendall, **sp. nov.**, *S.parvipilus* Jordal & Kirkendall, **sp. nov.**, *S.plenus* Jordal & Kirkendall, **sp. nov.**, *S.niger* Jordal & Kirkendall, **sp. nov.**, and *S.simplex* Jordal & Kirkendall, **sp. nov.** One species, *Scolytodesminutissimus* Schedl, 1952, is redescribed to match the holotype. We give new Costa Rica records for *S.costabilis* Wood, 1974, which is the correct name for *S.obesus* Wood, 1975 (**syn. nov**.). We report Costa Rica as a new country record for six species: *Scolytodesclusiacolens* Wood, 1967, *S.crinalis* Wood, 1978, *S.culcitatus* (Blandford, 1897), *S.libidus* Wood, 1978, *S.reticulatus* (Wood, 1961), and *S.spadix* (Blackman, 1943). From a closely related genus, we provide the first record for Central America (and only the second collection) of *Pycnarthrumfulgidum* Wood, 1977.

## Introduction

Modern biodiversity surveys of tropical arthropod faunas are discovering large numbers of new species and making them available to taxonomists. Good taxonomy, in turn, provides the cornerstones of biodiversity analyses. The Arthropods of La Selva projects (ALAS), supported by four NSF grants, ran from 1992 through 2005, with the aim of exhaustively sampling focal taxa in insects, mites and spiders from a Mesoamerican Neotropical forest (http://viceroy.eeb.uconn.edu/alas/history.html). The first three iterations (ALAS I–III) ran from 1992–2000 and concentrated on the lowland wet tropical forest of La Selva Biological Station run by the Organization for Tropical Studies on the Caribbean side of Costa Rica. ALAS IV was a five-year, 2000 m elevational gradient running SSW from La Selva up the north-eastern side of Volcan Barva to near the peak. The ALAS projects used a variety of quantitative sampling methods to survey both flying arthropods and those in vegetation or the leaf litter, including canopy sampling by UV light traps and canopy fogging (ALAS I–III only). Data from the ALAS project have contributed not only to our knowledge of the composition of hyperdiverse tropical arthropod communities but to ecological analyses of tropical biodiversity patterns as well (e.g., Longino and Colwell 1997, 2011; Colwell 2008; Kumar et al. 2011).

Kirkendall visited La Selva annually and participated in all the expeditions of the elevational gradient study (ALAS IV). He sampled bark beetles and pinhole borers by hand at all sites and has been identifying the large number of Scolytinae and Platypodinae collected. Many new species of these two weevil groups have been encountered in this material. Here, we describe 14 new species from *Scolytodes* Ferrari, 1867 that were collected during the ALAS projects, plus four other new species from Costa Rica.

*Scolytodes* Ferrari, 1867, is a highly diverse group of bark beetles distributed in the Neotropics from Florida and Mexico in the north, to subtropical Argentina in the south. There are many species at both low and high altitude; several species have been collected at close to 4000 m above sea level (Jordal 2018). Currently, 240 species are recognized, including the 18 new species described in this paper.

*Scolytodes* is of particular interest because of its wide range of breeding and feeding habits. Most species are typical monogynous bark beetles; a few species are harem polygynous (Wood 1982). Though many different host plants are recorded for *Scolytodes*, a strikingly high proportion breed in in *Cecropia* (Urticaceae) or *Clusia* (Clusiaceae) (Wood 1982; Jordal 1998b, 2013). Most species breed in inner bark, but several species groups reproduce in fallen leafstalks, some species breed in pith of twigs, and a few have only been found in vines and lianas. *Scolytodesunipunctatus* (Blandford, 1897) is an ambrosia beetle, whose larvae feed on symbiotic fungi growing in the parental tunnels in sapwood (Hulcr et al. 2007). Based on its atypical morphology, *S.culcitatus* (Blandford, 1897) could be a second, unrelated species with these habits (see below).

As is the case with many tropical bark beetles, most *Scolytodes* species are only known from a single collection and often only from a single specimen. Intensive arthropod field surveys in Neotropical forests have always found new species, especially those fogging canopy trees, but also from extracting insects from leaf litter, or using Malaise traps or flight intercept traps. *Scolytodes* is one of few scolytine genera that are found throughout the forest floor and canopy, in many host trees, and at all forested altitudes (Jordal 1998a, b, 2013, 2018).

## Material and methods

Most of the material treated in this paper is based on collections made during the ALAS projects. In addition to the low altitude location at La Selva Biological Station, ALAS IV included sites on Volcan Barva at 300 m, 500 m, 1000 m, 1500 m, and 2000 m, thus covering all major forest types in Braulio Carrillo National Park.

Material originally deposited in INBio were transferred several years ago to the National Museum of Costa Rica (MNCR) and most of the types designated in this paper therefore belong to MNCR. These and other specimens studied are deposited in the following institutions:

**CASC**California Academy of Sciences, San Francisco, California, USA.

**CMNC**Canadian Museum of Nature (Entomology Div.), Ottawa, Ontario, Canada.

**CNCI**Canadian National Collection of Insects, Ottawa.

**EMEC**Essig Museum of Entomology, University of California, Berkeley, California, USA.

**FSCA**Florida State Collection of Arthropods, Gainesville, Florida, USA.

**MNCR**Museo Nacional de Costa Rica, San José, Costa Rica.

**NHMW**Naturhistorische Museum, Vienna, Austria.

**USNM** United States National Museum, Washington D.C. (Smithsonian)

**ZMBN**University Museum of Bergen, Norway.

All holotypes (or equivalent, e.g., Eggers ‘types’) of *Scolytodes* from USNM, NHMW, CNCI and MNHN and have been examined. One type is presumably lost (Budapest: *S.columbianus*). Type material of new species published by Bright (2019) was not examined directly but these have photos of the elytra and sufficiently detailed descriptions.

Measurements and morphological terminology are as used in previous papers on the genus (Jordal 1998b, 2018). *Scolytodes* is here treated as masculine as originally proposed and later corroborated by Alonso-Zarazaga and Lyal (2009). All female-amended names in Wood (2007) were therefore rejected.

## Results

### Scolytodes

Taxon classificationAnimaliaColeopteraCurculionidae

Ferrari

http://zoobank.org/0745C155-3489-4576-8A9B-8CA2026EFEE2

#### Diagnosis.

*Scolytodes* is recognized by the sharply carinate lateral edges of the pronotum, separated procoxae, and protibiae that have two large lateral teeth near the distal end which appear unsocketed (but are actually socketed teeth embedded in cuticle; see fig. 8 in Jordal, 1998b). The eyes are entire or weakly sinuate, and the antennal funicle is 5- or 6-segmented (including the pedicel; see Wood 1982).

### Scolytodes angulus

Taxon classificationAnimaliaColeopteraCurculionidae

Jordal & Kirkendall sp. nov.

http://zoobank.org/24302CAD-3CF7-43CF-A093-C7D5A3FA8AA3

Figs 1 , 4 , 7

#### Type material.

**Holotype, female**: Costa Rica, Puntarena, Monteverde, Estacion Biologica Monteverde, 10°19'10"N, 84°48'57"W, 1730 m, 12.VI.2001, R. Anderson, montane forest litter, 2001-107D. Holotype is deposited in USNM.

#### Diagnosis.

Interstriae 10 sharply elevated to near apex; protibiae with an additional mesal socketed tooth near the mucro. Distinguished from the closely related *S.erineophilus* Wood, 1969, by the densely placed and confused setae on the sutural interstriae and on declivity, and by the acute posterior corners of the pronotum.

#### Description female.

Length 1.7 mm, 2.4 × as long as wide; color brown, with densely placed white setae. ***Head.*** Eyes weakly sinuate, separated above by 1.6 × their width. Frons generally flattened, slightly bulging on central half; surface on bulging area glabrous, impunctate, transversely wrinkled, around this area densely punctured; vestiture consisting of median long setae in a circle around bulging area, a few more short setae at upper level of eyes. Antennal club with two obliquely procurved sutures marked by setae, segments 1 and 2 corneous, segment 3 setose. Funiculus 6-segmented. ***Pronotum*** parallel-sided except distinctly expanded laterally in posterior corners; surface reticulate, dull, with shallow tiny punctures on basal fourth, on anterior three-fourths densely asperous. Vestiture consisting of fine erect and recumbent setae (4–0–0). ***Elytra*** smooth, striae weakly impressed, punctures small, deep, subcontiguous; interstriae approximately 3–5 × as wide as striae, punctures similar to those in striae, strongly confused or in rows. Interstriae 10 sharply elevated to near apex. Vestiture consisting of regular rows of long fine setae on each interstria, becoming spatulate on sutural interstriae, mixed with shorter fine hair-like setae in striae and interstriae. ***Legs.*** Procoxae separated by 1.0 × and mesocoxae 1.1 × the width of one procoxa. Protibiae very narrow, lateral teeth 1 and 2 sub-equal, with 3–4 additional tiny granules along the edge towards base; an additional mesal socketed tooth near the mucro; protibial mucro short, straight. Meso- and metatibiae with 6 socketed lateral teeth on distal half and third, respectively. ***Ventral vestiture.*** Setae on metanepisternum bifid or plumose, on metasternum simple.

#### Male.

Not known.

#### Key

(Wood 1982). Keys to couplet 41, *S.erineophilus*, but distinguished as noted in the diagnosis.

#### Etymology.

The Latin name *angulus* is a masculine noun in apposition meaning corner or angle, referring to the sharp hind corners of the pronotum.

#### Biology and distribution.

This species is only known from the type locality in Costa Rica, in high altitude cloud forest.

### Scolytodes niger

Taxon classificationAnimaliaColeopteraCurculionidae

Jordal & Kirkendall sp. nov.

http://zoobank.org/C160D713-112C-45F3-8FCB-B3CE555CBA7C

Figs 2 , 5 , 8

#### Type material.

**Holotype, female**: Costa Rica, Guanacaste prov., Rincon de La Vieja, Las Pailas, 18.II.1996-020A, R. Anderson, *Clusiarosea* forest litter. **Allotype male**: Costa Rica, Puntarenas, 11 km SW Est. Biol. Las Cruces, 1450 m, 08°46'43"N, 83°01'50"W, 9.VII.1999, R. Anderson, wet cloud forest litter, 99-124E. **Paratypes**: Prov. Heredia, 6 km ENE Vara Blanca, 1950–2050 m, 10°11'N, 84°07'W, 21 Feb. 2002, INBio–OET–ALAS transect, 20/TN/12/005 (INB0003223381) (1); San José prov., Zurquí de Moravia, 1600 m, 30.6.1997, L. Kirkendall, H. Lezama, flight intercept trap (1). Holotype and allotype deposited in CMNC, 1 paratype in MNCR, and 1 paratype in ZMBN.

#### Diagnosis.

Interstriae 10 carinate to level of ventrite 2; protibiae with an additional mesal tooth near tarsal insertion. Metanepisternum covered with white plumose setae. Very similar to *S.clusiae* Wood, 1969, but differs by the smooth and shiny pronotum, the shorter setae in a smaller area in the female frons, and by the distinct, albeit tiny, interstrial punctures.

#### Description female.

Length 1.5 mm, 2.1 × as long as wide; color black. ***Head.*** Eyes sinuate, separated above by 2.0 × their width. Frons weakly impressed from just above level of antennal insertion to epistoma; surface smooth, densely punctured in impressed area, scattered punctures above, shiny. Vestiture consisting of short setae in impressed area and on epistoma. Antennal club with two sutures marked by shorter setae, segment 1 and 2 corneous, sutures slightly constricted; funiculus 6-segmented. ***Pronotum*** shiny, with shallow, small, elongated punctures spaced by 2–3 × their diameter (length). Vestiture consisting of 8 erect long setae (4–2–2). ***Elytra*** generally smooth and shiny; striae not impressed, punctures shallow, tiny, appear elongated but composed by two punctures in one, each pair separated in rows by their length; interstriae 4 × as wide as striae, punctures of same size as in striae, much more separated, mainly in rows. Interstriae 10 carinate to level of ventrite 2. Vestiture consisting of regular rows of erect, interstrial setae of variable thickness, and regularly placed, fine, short, semi-recumbent setae in striae and interstriae, mainly on posterior half. ***Legs.*** Procoxae broadly separated by 0.9 × and mesocoxae 1.2 × the width of one procoxa. Protibiae slightly broadening distally, lateral teeth 1 and 2 of similar size, with a faint extension of cuticle between them, and with 3–4 additional small, sharp spines or granules along the lateral edge towards base; an additional mesal tooth present near tarsal insertion; protibial mucro obtuse. Meso- and metatibiae with 5 and 6 lateral, socketed, small teeth on distal half and third, respectively. ***Ventral vestiture.*** Setae on metanepisternum quadrifid to strongly plumose and densely placed, on mesanepisternum mainly trifid, on metasternum mainly simple, some bifid. Sclerolepidia scale-like or plumose.

#### Male.

1.4–1.6 mm long, 2.0–2.1 × as wide as long. Identical to female, except frons slightly more convex on lower part, with less dense vestiture.

#### Key

(Wood 1982). Keys to couplet 25 with some uncertainty; *S.clusiae* in couplet 24 is a better match.

#### Etymology.

The Latin name *niger* is a masculine adjective, meaning shining black, referring to the dark and shiny appearance of this species.

#### Biology and distribution.

This species is known from four Costa Rican cloud forest localities – in the northern and southern slopes of Volcan Barva (Braulio Carrillo), the southern slope of Rincon de La Vieja, and close to the Panama border (Las Cruces). Two specimens were collected by flight intercept trapping, and two by leaf litter sifting, one of which from *Clusia* litter.

### Scolytodes simplex

Taxon classificationAnimaliaColeopteraCurculionidae

Jordal & Kirkendall sp. nov.

http://zoobank.org/7BFB9FF3-22E2-4529-B25C-A12FD0268B99

Figs 3 , 6 , 9

#### Type material.

**Holotype, female**: Costa Rica, Cartago, km 89 PanAmHighway, Cerro de la Muerte, 3300 m, 10.II.1996-002A, R. Anderson, elfin bamboo forest litter. **Paratype**: same data as holotype, except Cerro Buenavista, 3200 m, 09°33'N, 83°45'30"W, 18.VI.1998, R. Anderson, elfin bamboo/ mixed subparamo litter 98-102D (1). Holotype and paratype deposited in USNM.

#### Diagnosis.

Interstriae 10 carinate to level of metacoxae; protibiae broad with an additional mesal tooth near base of tooth 2. Similar to *S.venustulus* Wood, but is much larger, with shiny pronotum punctured to anterior margin, and finer elytral setae. Also rather similar to *S.radiatus* Wood, 1977, but differs by having much smaller punctures on pronotum and elytral striae, and the presence of setae in the female frons. Differs from *S.clusiae* Wood, 1969 by the smooth and shiny pronotum, the female frons is less setose without impressed area, and by the distinct, albeit tiny, interstrial punctures.

#### Description female.

Length 1.9–2.0 mm, 2.1–2.2 × as long as wide; color black. ***Head.*** Eyes entire, separated above by 2.9–3.0 × their width. Frons convex, slightly bulging near upper level of eyes, flat below and level with epistoma; surface smooth, densely punctured on median half from just above level of antennal insertion to epistoma, shiny and impunctate above, with scattered punctures on vertex. Vestiture consisting of scant short fine setae on lower half. Antennal club with two slightly procurved sutures marked by short, coarse, setae, segment 1 and 2 rather large, corneous, suture 1 constricted; funiculus 5-segmented. ***Pronotum*** shiny, with deep, variably sized punctures spaced by 1–3 × their diameter. Vestiture consisting of 4 long, semi-erect, fine setae (4–0–0) and some fine, recumbent, setae along the anterior margin. ***Elytra*** generally smooth and shiny; striae not impressed, punctures shallow, tiny, appears elongated but composed by two punctures in one, each pair separated in rows by less than their length; interstriae 4 × as wide as striae, punctures of same size as in striae, much more and irregularly separated, mainly in rows. Interstriae 10 carinate to level of metacoxae. Vestiture consisting of about 20 erect setae of variable length on odd-numbered interstriae, and minute, recumbent setae in striae and interstriae. ***Legs.*** Procoxae separated by 0.5 × and mesocoxae 1.0 × the width of one procoxa. Protibiae broad distally, lateral teeth 1 and 2 of similar size, tooth 2 exposed, socketed, with 4–6 additional small, sharp spines or granules along the lateral edge towards base; an additional mesal tooth present near base of tooth 2; protibial mucro curved posterio-laterally. Meso- and metatibiae with 5 and 6 lateral, socketed teeth on distal half and third, respectively. ***Ventral vestiture.*** Setae on metanepisternum and metasternum simple, with some occasional bifid setae; on mesanepisternum strongly plumose. Sclerolepidia large, scale-like.

#### Male.

Presumably similar to the female.

#### Key

(Wood 1982). Keys to couplet 25a, *S.radiatus*, but differs as noted in the diagnosis.

#### Etymology.

The Latin name *simplex* is an invariable adjective meaning plain or simple, in the sense of lacking ornaments, referring to the ordinary female frons.

#### Biology and distribution.

This species is only known from two nearby locations at very high altitude in Costa Rica. Two specimens were sifted from mixed bamboo and elfin forest litter.

**Figures 1–9.**
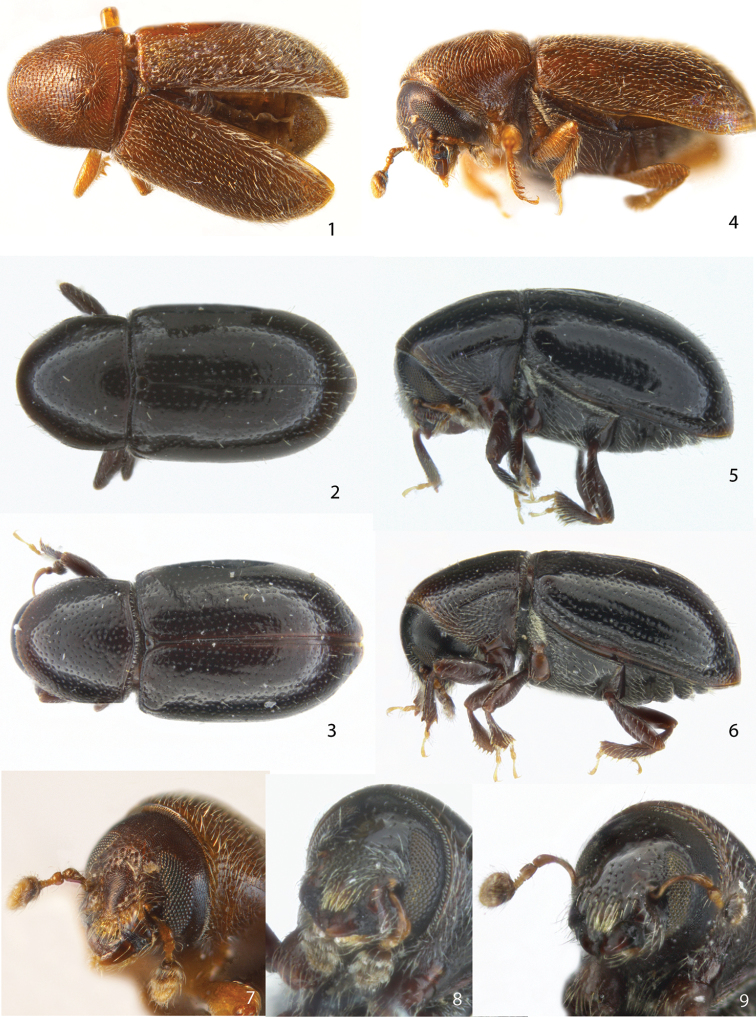
Dorsal, lateral and frontal view of the female holotype of **1, 4, 7***S.angulus*, **2, 5, 8***S.niger* and **3, 6, 9***S.simplex*.

### Scolytodes sufflatus

Taxon classificationAnimaliaColeopteraCurculionidae

Jordal & Kirkendall sp. nov.

http://zoobank.org/83A5494B-EA63-4D05-B09E-A689400CDDBE

Figs 10 , 13 , 16

#### Type material.

**Holotype female**: Costa Rica, Prov. Heredia, 6 km ENE Vara Blanca, 1950–2050 m, 10°11'N, 84°07'W, 21 Apr. 2002, INBio-OET-ALAS transect, 20/TN/17/030, INB0003223002. **Allotype male**: Alajuela, PN Volcan Poas, 2500 m, 10°11'30"N, 84°14'W, 6.VI.1997, R. Anderson, wet cloud forest litter, RSA 1997-003E. **Paratypes**: same data as holotype, except 22 Mar., 20/TN/08/011, INB0003222886 (1); San José, km 72, Int. Amer. Hwy, 3 km W. Ojo de Agua, 2950 m, 9°37'30"N, 83°50'30"W, 7.VI.1997, R. Anderson, oak for. Litter, 97-005B (1). Holotype and allotype in MNCR, 1 paratype in USNM, 1 paratype in ZMBN.

#### Diagnosis.

Interstriae 10 sharply elevated to level of metacoxae, interstriae 9 sharply elevated from near base to near apex; protibiae with an additional socketed, thin tooth near the mucro, posterior surface of protibia sulcate and strongly reticulate. Distinguished from *S.radiatus* by the much smaller punctures on the pronotum, by the paired smaller punctures in elytral striae, and a much longer carinate interstriae 9.

#### Description female.

Length 1.7–1.9 mm, 2.2–2.4 × as long as wide; color black. ***Head.*** Eyes entire, separated above by 3.1–3.3 × their width. Frons bulging on lower two-thirds, transition to epistoma smooth; surface smooth and shiny, largely impunctate; vestiture consisting of 6–8 scattered long setae near epistoma and eyes. Antennal club with two obliquely procurved sutures densely marked by setae, corneous part of segments 1 and 2 barely visible. Funiculus 5-segmented. ***Pronotum*** smooth, strongly reticulate, dull, with scattered faint punctures reaching anterior margin. Vestiture consisting of two fine erect median setae along anterior margin (2–0–0), otherwise glabrous. ***Elytra*** smooth, striae 1 weakly, others not impressed, punctures small, in pairs of two subcontiguous punctures, each pair spaced by the length of each pair; interstriae 4–5 × as wide as striae, single punctures spaced by 5–10 × their diameter, in rows. Interstriae 10 sharply elevated to level of metacoxae; interstriae 9 sharply elevated from near base to near apex. Vestiture consisting of 10 erect long very fine setae, three on each interstria 3, two on each interstria 7. ***Legs.*** Procoxae separated by 0.4 × and mesocoxae 0.8 × the width of one procoxa. Protibiae broad, posterior surface of protibia sulcate and strongly reticulate; lateral teeth 1 and 2 of sub-equal size, cuticle extending around these, with 3 additional tiny granules along the edge towards base; an additional socketed, thin tooth present near the mucro; protibial mucro curved posteriorly. Meso- and metatibiae with 6–7 thin socketed lateral teeth on distal half and third, respectively. ***Ventral vestiture.*** Scattered setae on metanepisternum and metasternum long and simple; sclerolepidia large round scales.

#### Male.

Identical to female. The terminal tergite of two paratypes were examined and revealed separate tergites 7 and 8 (male), and a single broader tergite 7 (female).

#### Key

(Wood 1982). Keys to couplet 23, but punctures on elytral declivity do not match. If not, it will key to couplet 25a, *S.radiatus*.

#### Etymology.

The name *sufflatus* is a Latin masculine participle, meaning puffed up or inflated, referring to the swollen frons in both sexes.

#### Biology and distribution.

This species is known from three localities above 2000 m altitude in Costa Rica.

### Scolytodes squamatifrons

Taxon classificationAnimaliaColeopteraCurculionidae

Jordal & Kirkendall sp. nov.

http://zoobank.org/B56E6C09-F0EB-4302-8BAA-D799C323B189

Figs 11 , 14 , 17

#### Type material.

**Holotype, female**: Costa Rica, San José, km 68 PanAmHighway, Tres de Junio Bog, 2600 m, 10.I.1996-001C, R. Anderson, litter ex. Forest adjacent to *Sphagnum* bog. Holotype deposited in USNM.

#### Diagnosis.

Interstriae 10 sharply elevated to level of metacoxa, interstriae 9 sharply elevated from the level of metacoxa to near apex; protibiae with an additional socketed, thin tooth near the mucro, posterior surface of protibiae sulcate and strongly reticulate. Distinguished from *S.sufflatus* by the impressed female frons, the broader eyes which are more closely situated above, by the narrower shape of the body, the shorter elytral setae, and by the shorter carinate part of interstria 9.

#### Description female.

Length 1.5 mm, 2.4 × as long as wide; color black. ***Head.*** Eyes entire, separated above by 2.3 × their width. Frons generally flat, circularly impressed on median half; surface around impression smooth and shiny, impression with densely set punctures and tiny granules; vestiture in impressed area consisting of densely placed short scale-like setae, on epistoma and near antennal insertion with scant hair-like setae. Antennal club setose, with two obliquely procurved sutures barely discernible. Funiculus 5-segmented. ***Pronotum*** smooth, strongly reticulate, with scattered faint punctures reaching anterior margin. Vestiture consisting of two fine erect median setae along anterior margin (2–0–0), otherwise glabrous. ***Elytra*** smooth, striae 1 weakly, others not impressed, punctures small, in pairs of two subcontiguous punctures, each pair spaced by the length of each pair; interstriae 4–5 × as wide as striae, single punctures spaced by 5–10 × their diameter, in rows. Interstriae 10 sharply elevated to level of metacoxa, interstriae 9 sharply elevated from the level of metacoxa to near apex. Vestiture consisting of 4–6 short erect very fine setae, mainly on interstriae 3. ***Legs.*** Procoxae separated by 0.4 × and mesocoxae 0.8 × the width of one procoxa. Protibiae broad, posterior surface sulcate and strongly reticulate, lateral teeth 1 and 2 of sub-equal size, with cuticle extending between these, with 3 additional tiny granules along the edge towards base; an additional socketed, thin tooth present near the mucro; protibial mucro curved posteriorly. Meso- and metatibiae with 6–7 fine socketed lateral teeth on distal half and third, respectively. ***Ventral vestiture.*** Scattered setae on metanepisternum and metasternum long and simple; sclerolepidia large round scales.

#### Male.

Unknown.

#### Key

(Wood 1982). As for *S.sufflatus*.

#### Etymology.

The name *squamatifrons* is composed by the stem of the Latin adjective *squamatus*, meaning scaly, a linking vowel –i, and the noun *frons*, meaning forehead. It is invariable.

#### Biology and distribution.

This species is known from the type locality at high altitude in Costa Rica.

### Scolytodes comosus

Taxon classificationAnimaliaColeopteraCurculionidae

Jordal & Kirkendall sp. nov.

http://zoobank.org/9575F9F8-16CE-45B0-946D-CE371D965F4E

Figs 12 , 15 , 18

#### Type material.

**Holotype**: Costa Rica, Prov. Heredia, 9 km ENE Vara Blanca, 1450–1550 m, 10°14'N, 84°06'W, 11 Apr. 2005, INBio-OET-ALAS transect, ex *Cecropia* petiole, 050411. **Paratypes**: Same data as holotype (4); Alajuela Res. For. San Ramon, 25 km NE San Ramon, 20 Nov. 1993, L.R. Kirkendall, ex *Cecropiapeltata* petiole (1). Holotype and 2 paratypes deposited in MNCR, 1 paratype in ZMBM, 2 in USNM.

#### Diagnosis.

Interstriae 10 not elevated; protibiae without additional mesal tooth. Setae on pronotum, and elytral striae and interstriae very long. Distinguished from *S.punctiferus* Wood, 1976 and *S.punctatus* (Eggers, 1943) by the longer setae, by the broader body shape and more broadly separated eyes.

#### Description male and female.

Length 1.2–1.4 mm, 1.9 × as long as wide; mature color dark brown. ***Head.*** Eyes entire, separated above by 2.8 × their width. Frons generally flat, surface impunctate and shiny on median fifth from epistoma to vertex, scattered deep punctures along this line; vestiture consisting of sparse long fine setae in punctured area, pointing medially. Antennal club setose, sutures not discernible, basal part of segment 1 corneous. Funiculus 5-segmented. ***Pronotum*** smooth, weakly reticulate, subshining, with deep large punctures reaching anterior margin, spaced on average by their diameter. Vestiture consisting of very long fine setae (0–0–0). ***Elytra*** smooth, striae not impressed, punctures large, deep, spaced by their diameter; interstriae difficult to distinguish from striae, occasionally in rows but largely confused with striae; all punctures on sides slightly raised on anterior margin, appearing granulated in dorsal profile. Interstriae 10 not raised. Vestiture consisting of very long fine setae on interstriae and striae. ***Legs.*** Procoxae separated by 1.0 × and mesocoxae 1.5 × the width of one procoxa. Protibiae broad, lateral teeth 1 and 2 of sub-equal size, tooth 2 exposed, both teeth extended distally, with a sharp serrated edge running from tooth 2 towards base; protibial mucro large, curved posterior-distally. Meso- and metatibiae with 6 and 7 coarse socketed lateral teeth on distal two-thirds and half, respectively. ***Ventral vestiture.*** Setae on metanepisternum bifid, on metasternum long and simple; sclerolepidia and setae on epimeron plumose.

#### Key

(Wood 1982). Keys incorrectly to couplet 7 due to the mesal tooth on protibiae is missing. The same applies to *S.punctiferus*, which is nevertheless placed in couplet 20.

#### Etymology.

The name *comosus* is a Latin masculine adjective, meaning long haired, referring to the confused rows of long strial, interstrial, and pronotal setae.

#### Biology and distribution.

This species is known from two median altitude localities near Volcan Poas in Costa Rica. It was taken from *Cecropia* petioles in both sites. The species was recorded as *Scolytodes* sp. B by Jordal and Kirkendall (1998).

**Figures 10–18.**
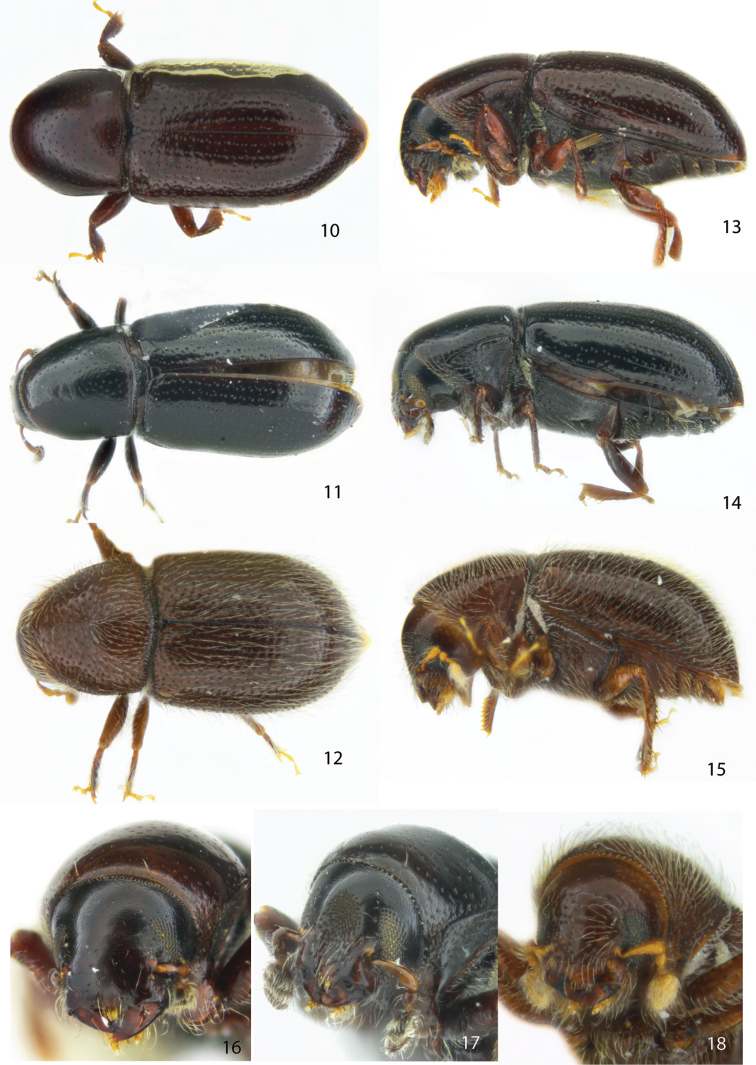
Dorsal, lateral and frontal view of the female holotype of **10, 13, 16***Scolytodessufflatus***11, 14, 17***S.squamatifrons***12, 15, 18***S.comosus*.

### Scolytodes spatulatus

Taxon classificationAnimaliaColeopteraCurculionidae

Jordal & Kirkendall sp. nov.

http://zoobank.org/D4ABB6CF-6F5B-463F-A351-3D1E24D1A676

Figs 19 , 22 , 25

#### Type material.

**Holotype**: Costa Rica, Prov. Heredia, 11 km ESE La Virgen, 250–350 m, 10°21'N, 84°03'W, 21 Mar. 2004, INBio-OET-ALAS transect, Intercept trap 03/TN/14/017, INB0003619017. Holotype deposited in MNCR.

#### Diagnosis.

Interstriae 10 weakly carinate to level of metacoxa, interstriae 9 carinate on posterior half; protibiae without additional mesal tooth. Distinguished from *S.comosus* by the long spatulate setae on each declivital striae, by the broader shape of the pronotum, the more narrowly separated eyes, and by the plumose setae on the metanepisternum.

#### Description, female(?)

Length 1.3 mm, 2.1 × as long as wide; color dark brown. ***Head.*** Eyes entire, separated above by 2.1 × their width. Frons generally flat, narrowly impressed just above epistoma, surface irregularly punctured, reticulate; vestiture consisting of sparse long fine setae pointing medially. Antennal club setose, sutures not discernible, basal part of segment 1 corneous. Funiculus possibly 5-segmented. ***Pronotum*** smooth on posterior half, with shallow punctures spaced by 1–2 × their diameter, on anterior half replaced by low scattered asperities; surface reticulate, subshining. Vestiture consisting of long fine setae (0–0–0). ***Elytra*** generally smooth, but punctures crenulated; striae not impressed, punctures small, crenulated on the anterior margin, spaced by 1–2 × their diameter; punctures in interstriae similar and entirely confused with those in striae. Interstriae 10 weakly carinate to level of metacoxa, interstriae 9 carinate on posterior half. Vestiture consisting of long fine strial and interstrial setae, with additional spatulate long setae in each striae on declivity. ***Legs.*** Procoxae separated by 1.0 × and mesocoxae 1.2 × the width of one procoxa. Protibiae broad, lateral tooth 1 as long as 2, tooth 2 exposed and socketed, with additional 2–3 granules along the lateral edge towards base; protibial mucro large, and curved posteriorly. Meso- and metatibiae with 6 socketed lateral teeth on distal half. ***Ventral vestiture.*** Setae on metanepisternum trifid or plumose, on metasternum long and simple; sclerolepidia and setae on epimeron plumose.

#### Key

(Wood 1982). Keys to couplet 6 with no match (see *S.comosus*, above). One should reach couplet 20, near *S.punctiferus*.

#### Etymology.

The Latin name *spatulatus* is a masculine adjective, meaning spatula-like, referring to the broad tips of the longest setae on declivity.

#### Biology and distribution.

This species is only known from the type locality in a lowland rain forest site south of La Selva Biological Station.

### Scolytodes seriatus

Taxon classificationAnimaliaColeopteraCurculionidae

Jordal & Kirkendall sp. nov.

http://zoobank.org/5CC0C3CB-F4C7-48C4-8B32-E2E735E86068

Figs 20 , 23 , 26

#### Type material.

**Holotype, female**: Costa Rica, Prov. Heredia, 6 km ENE Vara Blanca, 1950–2050 m, 10°11'N, 84°07'W, 21 Feb. 2002, INBio-OET-ALAS transect, 20/TN/15/008, INB0003223280. **Allotype, male**: same data as holotype, except 9 Apr., transect 20/M/06/066, INB0003221635. **Paratypes**: same data as holotype except 21 Apr., transect 20/TN/15/028, INB0003223644 (1); 13 Apr., transect 20/RG/DBM/010, INB0003657266 (1). Holotype and allotype deposited in MNCR, 1 paratype in USNM, 1 paratype in ZMBN.

#### Diagnosis.

Interstriae 10 sharply carinate to near apex; protibiae with a tiny, sharp, additional mesal tooth near tarsal insertion. Distinguished from *S.clusiacolens* Wood, 1967 and *S.prolatus* Jordal, 2018 by the stouter body shape and subobovate elytra, by the smaller body size, and by the regular placement of fine setae only on interstriae 3, 5, 7 and 9.

#### Description female.

Length 2.1–2.3 mm, 2.3–2.4 × as long as wide; color black. ***Head.*** Eyes entire, separated above by 2.5–3.0 × their width. Frons impressed on a semi-circular area from just below upper level of eyes to epistoma, surface finely, densely punctured, finely reticulate; vestiture consisting of fine short setae. Antennal club with two obliquely procurved sutures marked by white setae, segment 1 and 2 mainly corneous, segment 3 setose. Funiculus 6-segmented. ***Pronotum*** smooth, finely reticulate, with fine punctures spaced by 1–2 × their diameter. Vestiture consisting of six erect long setae (4–2–0). ***Elytra*** smooth, shiny, striae distinctly impressed, punctures in rows spaced by 2 × their diameter; punctures in interstriae in irregular rows, shallow, smaller and more widely spaced. Interstriae 10 sharply carinate to near apex. Vestiture consisting of erect long setae, on interstriae 3, 5, 7 and 9. ***Legs.*** Procoxae separated by 0.5 × and mesocoxae 0.9 × the width of one procoxa. Protibiae narrow, lateral teeth 1 and 2 of sub-equal length, with 4–5 additional sharp small teeth along the lateral edge towards base; a tiny, sharp, additional mesal tooth present near tarsal insertion; protibial mucro short and curved, almost obtuse. Meso- and metatibiae with 6–7 small socketed lateral teeth on distal half and third, respectively. ***Ventral vestiture.*** Setae on metanepisternum trifid or bifid, on metasternum long and simple; sclerolepidia rounded scales.

#### Male.

Similar to female except frons convex, with distinct abrupt impression on epistoma; eyes separated above by 3.3–3.6 × width of the eye; vestiture on frons consisting of few short setae.

#### Key

(Wood 1982). Keys to couplet 6 but do not match due to the combination of a long interstriae 10 and the presence of a mesal protibial tooth. The same applies to *S.clusiacolens*, its closest relative.

#### Etymology.

The Latin name *seriatus* is a Latin masculine adjective meaning seriate, referring to the deeply impressed rows of closely set punctures in the striae.

#### Biology and distribution.

This species is only known from the type locality in Costa Rica at high altitude.

### Scolytodes profundus

Taxon classificationAnimaliaColeopteraCurculionidae

Jordal & Kirkendall sp. nov.

http://zoobank.org/EE501239-19BC-43E8-951F-EA32A31F2EB8

Figs 21 , 24 , 27

#### Type material.

**Holotype, female**: Costa Rica, Guanacaste, Guanacaste cons. Area, Rincon de La Vieja, Las Pailas, 1650 m, 19.II.1996-021A, R. Anderson, windblown cloud forest ridge litter. **Allotype, male**: same data as holotype. **Paratypes**: Alajuela, PN Volcan Poas, 2500 m, 10°11'30"N, 84°14'W, 6.VI.1997, R. Anderson, wet cloud for. litter, 97-003 (4); wet mount. for. FIT 6-28.VI.97, S. & J. Peck, 97-16 (1); Hered. Prov, Cerro Chompipe, 2100 m 10km NNE Heredia, 12–27.VI.97, mont. for. FIT, S. & J. Peck, 97-21 (1); Prov. Heredia, 6 km ENE Vara Blanca, 1950–2050 m, 10°11'N, 84°07'W, 16 Mar. 2002, INBio-OET-ALAS transect, 20/WF/03/43, INB0003223948 (1); holotype, allotype and 1 paratype in FSCA, 2 paratypes in USNM, 2 paratypes in ZMBN, 2 paratypes in MNCR.

#### Diagnosis.

Interstriae 10 sharply carinate to near apex; protibiae with a tiny, additional mesal tooth at the base of lateral tooth 2. Distinguished from *S.seriatus* by the more elongated shape and narrowly rounded apex of the elytra, by the reticulate pronotum that is constricted on basal one-fourth, and the different female frons.

#### Description female.

Length 2.0–2.2 mm, 2.2–2.4 × as long as wide; color black. ***Head.*** Eyes entire, separated above by 3.3–3.6 × their width. Frons impressed on a semi-circular area from just below upper level of eyes to epistoma, surface densely punctured, except for a longitudinal median impunctate shiny glabrous band; vestiture consisting of fine short setae in punctured part of impressed area. Antennal club with two obliquely procurved sutures marked by short white setae, segment 1 and 2 mainly corneous, segment 3 setose. Funiculus 6-segmented. ***Pronotum*** slightly constricted laterally on basal on-fourth, surface smooth, reticulate, with fine punctures spaced by 2–3 × their diameter. Vestiture consisting of six erect long setae (4–2–0). ***Elytra*** smooth, shiny, striae distinctly impressed, punctures spaced by 2–3 × their diameter; punctures in interstriae confused, smaller and more widely spaced, intermixed with strial punctures. Interstriae 10 sharply carinate to near apex. Vestiture consisting of erect long acuminate setae on interstriae 3, 5, 7 and 9 only. ***Legs.*** Procoxae separated by 0.6 × and mesocoxae 0.9 × the width of one procoxa. Protibiae narrow, lateral teeth 1 and 2 sub-equal in length, with 5 small granules along the lateral edge towards base; a tiny, additional mesal tooth present at the base of lateral tooth 2; protibial mucro obtuse. Meso- and metatibiae with 6 small socketed lateral teeth on distal half and distal third, respectively. ***Ventral vestiture.*** Setae on metanepisternum bifid, on metasternum long and simple; sclerolepidia small rounded scales.

#### Male.

Similar to female, except frons convex, with distinct abrupt impression on epistoma; eyes separated above by 3.9–4.1 × the width of eye; vestiture of frons consisting of a few short setae.

#### Key

(Wood 1982). Keys to couplet 6 but does not match (long interstriae 10, presence of mesal protibial tooth). The same applies to *S.clusiacolens* and *S.seriatus*, presumably the closest relatives.

#### Etymology.

The Latin name *profundus* is a masculine adjective, meaning deep or profound, referring to the deeply impressed striae comprising relatively small but densely placed punctures.

#### Biology and distribution.

This species is known from several upper cloud forest localities in Costa Rica. One specimen was reared from sifted litter samples (miniWinkler method).

**Figures 19–27.**
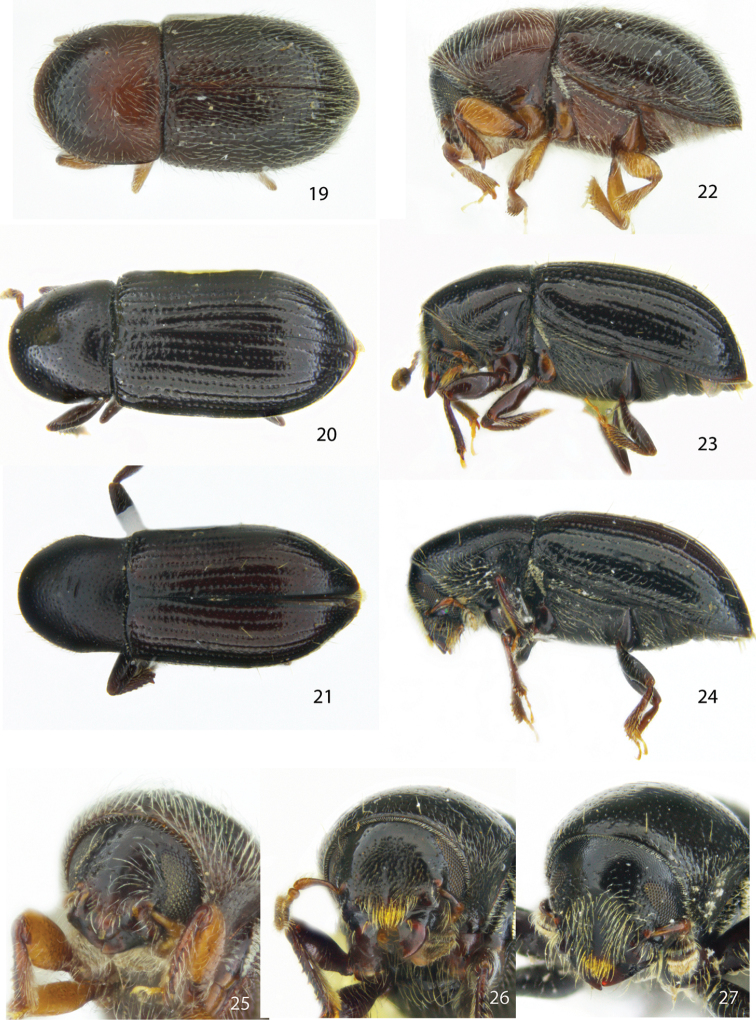
Dorsal, lateral and frontal view of the female holotype of **19, 22, 25***Scolytodesspatulatus***20, 23, 26***S.seriatus***21, 24, 27***S.profundus*.

### Scolytodes catinus

Taxon classificationAnimaliaColeopteraCurculionidae

Jordal & Kirkendall sp. nov.

http://zoobank.org/91F7BCD1-0E37-4B71-A04B-624043DE576D

Figs 28 , 31 , 34

#### Type material.

**Holotype, female**: Costa Rica, Heredia, near Virgen de Socorro, 10°14.4'N, 84°07.5'W, 1475 m, 11 Apr. 2005, L. Kirkendall #050411-1, 8 mm diameter *Clusia* branch. **Allotype, male**: same data as holotype. **Paratypes**: same data as holotype (3). Holotype, allotype, 1 paratype deposited in MNCR, 1 paratype in ZMBN, 1 paratype in USNM.

#### Diagnosis.

Interstriae 10 weakly carinate to just before level of metacoxa; protibiae with a tiny, additional mesal tooth at the base of lateral tooth 2. Pronotum asperate on anterior third. Metanepisternum with a row of characteristic, broad, plumose setae. Distinguished from *S.pumilus* Wood, 1969, by the lack of interstrial punctures, less abundant vestiture of the female frons, and the different setae on the metanepisternum.

#### Description female.

Length 1.7–1.8 mm, 2.6–2.7 × as long as wide; color black. ***Head.*** Eyes sinuate, separated above by 2.5–2.6 × their width. Frons weakly impressed on a semi-circular area on median half on lower two-thirds, surface in impressed area punctured, reticulate, dull, vertex shiny, with scant fine punctures; vestiture consisting of fine short setae in impressed area. Antennal club with two obliquely recurved sutures (posterior side procurved) marked by white setae, segment 1 and 2 corneous. Funiculus 5-segmented, segments very short, disc-like. ***Pronotum*** reticulate, with fine punctures on posterior two-thirds spaced by 3–4 × their diameter, replaced on anterior one-third by fine asperities. Vestiture consisting of six erect long setae (4–2–0). ***Elytra*** smooth, shiny, striae not impressed, punctures tiny, spaced by 2–3 × their diameter, with additional microscopic puncture associated near each main puncture; punctures in interstriae absent. Interstriae 10 weakly carinate to just before level of metacoxa. Vestiture consisting of ca 20 erect long setae on odd-numbered interstriae. ***Legs.*** Procoxae separated by 0.8 × and mesocoxae 1.1 × the width of one procoxa. Protibiae broadening apically, lateral teeth 1 and 2 of sub-equal size, with 3–6 additional small granules along the lateral edge towards base; a tiny, additional mesal tooth present at the base of lateral tooth 2; protibial mucro obtuse. Meso- and metatibiae with 6 and 5 small socketed lateral teeth on distal half and third, respectively. ***Ventral vestiture.*** Setae on metanepisternum broadly plumose, on metasternum long and simple; sclerolepidia small, round scales.

#### Male.

Similar to female, except frons convex, with slight impression on epistoma; vestiture consisting of a few setae on epistoma.

#### Key

(Wood 1982). Keys to couplet 53, *S.pumilus*, but differs as indicated in the diagnosis.

#### Etymology.

The Latin name *catinus* is a masculine noun meaning plate or bowl, referring to the smoothly impressed female frons. It is invariable.

#### Biology and distribution.

This species is only known from the type locality at medium altitude in Costa Rica. Specimens were collected from a thin dead branch on a live *Clusia* tree along with individuals of the smaller species *S.callosus* Jordal and Kirkendall (described below).

### Scolytodes fimbriatus

Taxon classificationAnimaliaColeopteraCurculionidae

Jordal & Kirkendall sp. nov.

http://zoobank.org/28003000-7A86-4107-B674-A1102D8B7289

Figs 29 , 32 , 35

#### Type material.

**Holotype, female**: Costa Rica, Alajuela Province, N slope Volcan de Rincon, 2 km W. Dos Rios, 550 m, V-22-85. Blacklight. J.T. Doyen and P.A. Opler coll. [EMEC49554]. **Paratype female**: same data as holotype. The holotype is deposited in EMEC, 1 paratype in USNM.

#### Diagnosis.

Interstriae 10 carinate to near apex; protibiae with a tiny, additional mesal tooth near base of mucro. Pronotum lightly wrinkled on anterior third, punctured from base to anterior margin. Distinguished from the most similar species *S.puer* (Schedl, 1952), *S.frontoglabratus* (Schedl, 1935) and *S.amoenus* Wood, 1967 by the long shiny median field on the female frons, the presence of exactly 6 erect setae on elytra, and by the presence of a minute additional mesal tooth on the protibia.

#### Description female.

Length 2.0–2.1 mm, 2.2 × as long as wide; color light reddish brown to black, darkest on anterior half of pronotum, and near the elytral base and suture. ***Head.*** Eyes entire, separated above by 2.7 × their width. Frons weakly flattened between eyes, a longitudinal shiny impunctate area on median third on lower half, surrounded by fine medium-long curved setae. Antennal club setose, with two two strongly procurved sutures marked by longer setae, segment 1 and 2 darker, almost corneous. Funiculus 6-segmented. ***Pronotum*** reticulate, with fine punctures spaced by 1–2 × their diameter, anterior half with very fine asperities in front of each puncture. Vestiture consisting of six erect long setae (4–0–2). ***Elytra*** smooth, shiny; striae not impressed, punctures in row spaced by their diameter; interstriae broad, 4 × as wide as striae, punctures confused except almost seriate on interstriae 4. Interstriae 10 carinate to near apex. Vestiture consisting of 6 erect setae, one each at base of interstriae 7, one near declivity on interstriae 9 and 7. ***Legs.*** Procoxae separated by 0.6 × and mesocoxae 0.8 × the width of one procoxa. Protibiae parallel-sided, lateral teeth 1 and 2 of equal size, with 4 additional small granules along the lateral edge towards base; a tiny, additional mesal tooth present near base of mucro; protibial mucro very short curved posteriorly. Meso- and metatibiae with 5–6 small socketed lateral teeth on distal half. ***Ventral vestiture.*** Setae on metanepisternum and metasternum simple, on mesanepisternum trifid; sclerolepidia small round scales.

#### Male.

Not known.

#### Key

(Wood 1982). Keys to couplet 9, *S.amoenus*, but differs as indicated in the diagnosis.

#### Etymology.

The Latin name *fimbriatus* is a masculine adjective, meaning fringed, referring to the broad circle of golden erect setae in the female frons, surrounding a large impunctate and shining area.

#### Biology and distribution.

This species is only known from the lowland type locality in Costa Rica. Two individuals were attracted to UV light.

### Scolytodes sulcifrons

Taxon classificationAnimaliaColeopteraCurculionidae

Jordal & Kirkendall sp. nov.

http://zoobank.org/8F18E732-7AB0-4B0D-9597-7CD98E03541F

Figs 30 , 33 , 36

#### Type material.

**Holotype, female**: Costa Rica, Prov. Heredia, 10 km SE La Virgen, 450–550 m, 10°20'N, 84°05'W, 14 Mar. 2003, #030314-3 [L. Kirkendall, leg]. **Allotype male and paratypes (2)**: same data as holotype. Holotype and allotype deposited in MNCR, 1 paratype in USNM, 1 paratype in ZMBN.

#### Diagnosis.

Interstriae 10 carinate to level of metacoxae; interstriae 9 carinate between level of metanepisternum and elytral apex; protibiae with an additional mesal tooth near tarsal insertion. Distinguished from the very similar species *S.planifrons* Jordal & Kirkendall (described below) and *S.clusiapraelatus* Jordal, 2013 by the sulcate female frons, the presence of several more and much longer elytral setae, and by the regularly sized and spaced strial punctures.

#### Description female.

Length 1.3–1.4 mm, 2.0–2.1 × as long as wide; color dark brown to black. ***Head.*** Eyes entire, separated above by 2.5–2.6 × their width. Frons impressed on median third from just below upper level of eyes to epistoma, slightly elevated along margin of impressed area; impressed and elevated area with deep large punctures approximately in longitudinal rows. Vestiture consisting of sparse, fine setae associated with punctures. Antennal club setose, with two obliquely procurved sutures weakly marked by longer setae. Funiculus 5-segmented. ***Pronotum*** reticulate, with minute punctures spaced by 4–8 × their diameter. Vestiture consisting of two erect long setae along the median part of the anterior margin (2–0–0). ***Elytra*** smooth, shiny; striae not (or very weakly) impressed, small punctures spaced by 2–3 × their diameter; interstriae 3–4 × as wide as striae, scant punctures barely visible, confused. Interstriae 10 carinate to level of metacoxae; interstriae 9 carinate between level of metanepisternum and elytral apex. Vestiture consisting of 10–14 erect long setae on interstriae 3 and 7. ***Legs.*** Procoxae separated by 0.6 × and mesocoxae 1.0 × the width of one procoxa. Protibiae broadening slightly distally, lateral teeth 1 and 2 of sub-equal size, with 3–4 additional, small granules along the lateral edge towards base; all teeth, particularly 1 and 2 are connected by a thin, semi-transparent extension of the cuticle; an additional mesal tooth present near tarsal insertion; protibial mucro straight, short. Meso- and metatibiae with 7 and 6 lateral, socketed, small teeth on distal half and third, respectively. ***Ventral vestiture.*** Setae on metanepisternum and metasternum simple, on mesanepisternum trifid; sclerolepidia small, round scales.

#### Male.

As in female, except frons convex, with scant short setae near epistoma.

#### Key

(Wood 1982). Keys to couplet 22, with no further match.

#### Etymology.

The Latin name *sulcifrons* is composed by the stem of the masculine adjective *sulcus*, meaning furrow, a linking vowel –i, and the noun *frons*, meaning forehead, referring to the broad longitudinal furrow in the female frons. It is invariable.

#### Biology and distribution.

This species is only known from the lowland type locality in Costa Rica. Individuals were collected from the bark of a 9 cm-diameter standing dead tree that was covered with thick moss.

**Figures 28–36.**
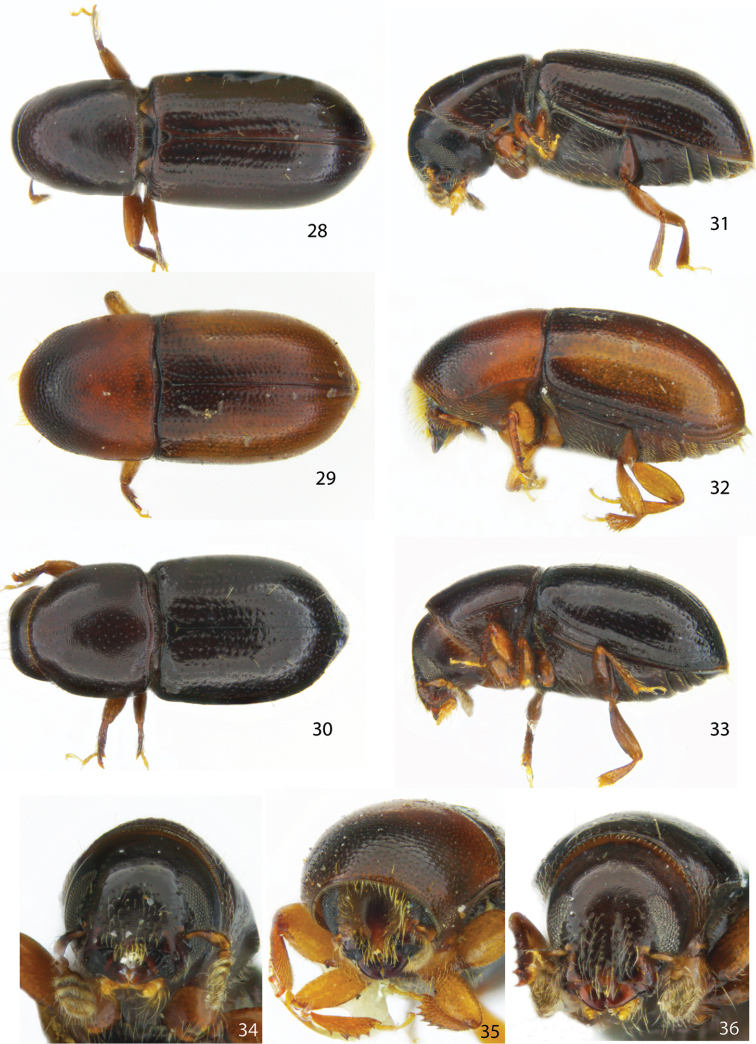
Dorsal, lateral and frontal view of the female holotype of **28, 31, 34***Scolytodescatinus***29, 32, 35***S.fimbriatus***30, 33, 36***S.sulcifrons*.

### Scolytodes planifrons

Taxon classificationAnimaliaColeopteraCurculionidae

Jordal & Kirkendall sp. nov.

http://zoobank.org/86558F26-7F63-4C40-BE9A-97AEB04C9897

Figs 37 , 40 , 43

#### Type material.

**Holotype, female**: Costa Rica, Prov. Heredia, 9 km NE Vara Blanca, 1450–1550 m, 10°14'N, 84°06'W, 22 Mar. 2005, INBio-OET-ALAS transect, Finca Murillo, 15/WF/01/50, INB0003665427. **Allotype male**: same data as holotype, INB0003665426. **Paratype**: same data as holotype, INB0003665425 (1f); 6 km ENE Vara Blanca, 1950–2050 m, 10°11'N, 84°07'W, 17 Feb. 2002, INBio-OET-ALAS transect, 20/WF/01/22, INB0003223047 (1f). Holotype and allotype deposited in MNCR, 1 paratype in USNM, 1 paratype in ZMBN.

#### Diagnosis.

Interstriae 10 carinate to level of metacoxae; interstriae 9 carinate between level of metanepisternum and elytral apex; protibiae with an additional mesal tooth near tarsal insertion. Distinguished from the the very similar species *S.sulcifrons* and *S.clusiapraelatus* Jordal, 2013 by the flat lower frons of females, the small elytral setae, and by the paired strial punctures.

#### Description female.

Length 1.4–1.5 mm, 2.0–2.1 × as long as wide; color dark brown to black. ***Head.*** Eyes entire, separated above by 2.8–3.0 × their width. Frons convex on upper half, entirely flat with scant fine punctures below. Vestiture consisting of sparse fine setae associated with punctures. Antennal club setose, with two transverse sutures weakly marked by longer setae. Funiculus 6-segmented. ***Pronotum*** shiny, very weakly reticulate, with small punctures spaced by 2–3 × their diameter. Vestiture consisting of two erect long setae along the median part of the anterior margin (2–0–0). ***Elytra*** smooth, shiny; striae not impressed, small punctures in rows, two and two in longitudinal pairs, each pair spaced by the length of a pair; interstriae 3–4 × as wide as striae, punctures of same size as in striae, widely spaced, mainly in rows. Interstriae 10 carinate to level of metacoxae; interstriae 9 carinate between level of metanepisternum and elytral apex. Vestiture consisting of 2–4 short erect interstrial setae, minute recumbent setae elsewhere. ***Legs.*** Procoxae separated by 0.5 × and mesocoxae 0.9 × the width of one procoxa. Protibiae broadening slightly distally, lateral teeth 1 and 2 of sub-equal size, with 2–3 additional small teeth along the lateral edge towards base; an additional mesal tooth present near tarsal insertion; protibial mucro, short, slightly curved posteriorly. Meso- and metatibiae with 7 and 6 small socketed lateral teeth on distal half. ***Ventral vestiture.*** Setae on metanepisternum and metasternum simple; sclerolepidia small round scales.

#### Male.

Nearly identical to female, frons slightly more rounded on lower half, body length 1.3–1.4 mm.

#### Key

(Wood 1982). Keys to couplet 22, with no further match.

#### Etymology.

The Latin name *planifrons* is composed by the stem of the masculine adjective *planus*, meaning flat, the linking vowel –i, and the noun *frons*, meaning forehead, referring to the completely flat and shiny lower frons of the female (and almost so in male). It is invariable.

#### Biology and distribution.

This species is only known from two high altitude localities in Costa Rica. The four individuals were reared from sifted litter samples (miniWinkler method).

### Scolytodes porosus

Taxon classificationAnimaliaColeopteraCurculionidae

Jordal & Kirkendall sp. nov.

http://zoobank.org/CEC1D42A-A771-4F6B-99C6-089CF14D080D

Figs 38 , 41 , 44

#### Type material.

**Holotype, presumably female**: Costa Rica, Prov. Heredia, 9 km NE Vara Blanca, 1450–1550 m, 10°14'N, 84°06'W, 6 Apr. 2005, INBio-OET-ALAS transect, #050406-3 [ex *Clusia*, L. Kirkendall, leg]. **Paratype** female: same data as holotype, except 8 Mar. 2005, 15/M/15/040, Finca Murillo, INB0003669571. Holotype deposited in MNCR, 1 paratype in USNM.

#### Diagnosis.

Interstriae 10 carinate to level of metacoxae; protibiae with an additional mesal tooth near tarsal insertion. Similar to *S.minutus* Wood, 1981, with the combination of deep large punctures on pronotum and elytra, and spatulate shape of elytral setae, but differs from *S.minutus* by the larger size, black color, and the smooth and more elongated pronotum.

#### Description female(?)

Length 1.5–1.6 mm, 2.7 × as long as wide; color dark brown to black. ***Head.*** Eyes entire, separated above by 2.6–2.7 × their width. Frons convex, with few shallow tiny punctures, surface shiny, reticulate on epistoma and vertex. Vestiture consisting of sparse fine setae on lower frons, denser on epistoma. Antennal club with two transverse sutures marked by short setae, segments 1 and 2 corneous, segment 3 setose. Funiculus 6-segmented. ***Pronotum*** shiny, with large deep punctures spaced by less than their diameter. Vestiture consisting of 8 longer erect setae (4–2–2), on the anterior part additional fine short setae. ***Elytra*** smooth, shiny; striae not impressed, punctures large, deep, separated in rows by less than their diameter, smaller on declivity; interstriae as broad as striae, punctures much smaller than in striae, widely spaced. Interstriae 10 carinate to level of metacoxae. Vestiture consisting of erect interstrial setae which are bristle-like near base of elytra and spatulate on posterior part and declivity, and fine short recumbent setae in striae. ***Legs.*** Procoxae separated by 0.4 × and mesocoxae 0.6 × the width of one procoxa. Protibiae broadening distally, lateral teeth 1 and 2 of equal size, tooth 2 socketed and exposed, with 2–3 additional small teeth along the lateral edge towards base; an additional mesal tooth present near tarsal insertion; protibial mucro obtuse. Meso- and metatibiae with 6 and 5 small socketed lateral teeth on distal half and third, respectively. ***Ventral vestiture.*** Setae on metanepisternum trifid to broadly plumose, on metasternum mainly simple, bifid near episternal suture; sclerolepidia broad plumose scales.

#### Male(?).

Presumably identical to the female. Sex of holotype is not determined but is identical to the female paratype with one elytron (exposing seven visible tergites).

#### Key

(Wood 1982). Keys to couplet 25, with no further match.

#### Etymology.

The Latin name *porosus* is a masculine adjective, meaning porous, referring to the densely and deeply punctured pronotum and elytra.

#### Biology and distribution.

This species is only known from the high altitude type locality in Costa Rica. One individual was collected in a Malaise trap, the other was dissected from a *Clusia* branch.

### Scolytodes mundus

Taxon classificationAnimaliaColeopteraCurculionidae

Jordal & Kirkendall sp. nov.

http://zoobank.org/AD4DA531-187D-4450-A4B1-959D69F1001D

Figs 39 , 42 , 45

#### Type material.

**Holotype, female**: Costa Rica, Prov. Heredia, 11 km SE La Virgen, 450–550 m, 10°20'N, 84°05'W, 17 Mar. 2003, INBio-OET-ALAS transect, 05/F/02/30, INB0003605900. **Allotype, male**: same data as holotype, 05/F/02/30 (INB0003605847). **Paratypes**: same data as holotype, 05/F02/13 (INB0003605727) (1); same data except 16 Feb., 05/F/01/24 (1); same data except 12 Apr., 05/F/03/37 (INB0003605900) (1). Holotype and 2 paratypes are deposited in MNCR, 1 paratype in ZMBN, 1 in USNM.

#### Diagnosis.

Interstriae 10 carinate to level of ventrite 1; protibiae with an additional mesal tooth near base of tooth 2. Distinguished from the very similar *S.callosus* Jordal & Kirkendall (described below) by the convex female frons which lacks a callus above epistoma, by the abundance of confused interstrial micro-punctures, and the entire eyes which are more broadly separated above.

#### Description, female.

Length 1.3–1.5 mm, 2.7 × as long as wide; color black. ***Head.*** Eyes entire, separated above by 2.8–3.1 × their width. Frons convex, short; surface weakly reticulate, more strongly so on vertex, with large punctures intermixed with micro-punctures on lower half. Vestiture consisting of fine setae from below upper level of eyes to epistoma. Antennal club and funiculus not clearly visible on specimens. ***Pronotum*** shiny, with shallow small punctures spaced by 3–4 × their diameter; faint asperities present on anterior fifth. Vestiture consisting of 8 erect long setae (4–2–2). ***Elytra*** smooth and shiny, cuticle slightly wrinkled at interstriae 1 and 2; striae 1 and 2 weakly impressed, others not, punctures small, deep, associated with a micro-puncture and together appears like one elongated puncture, each pair separated in rows by less than their length; interstriae 3–4 × as wide as striae, with abundant, confused micro-punctures, particularly abundant on interstriae 1–5. Interstriae 10 carinate to level of ventrite 1. Vestiture consisting of about 40 erect setae regularly distributed on odd-numbered interstriae. ***Legs.*** Procoxae separated by 0.4 × and mesocoxae 0.9 × the width of one procoxa. Protibiae narrow, lateral teeth 1 and 2 of equal size, with 3 additional small teeth along the lateral edge towards base; an additional mesal tooth present near base of tooth 2; protibial mucro obtuse. Meso- and metatibiae with 6 and 5 small socketed lateral teeth on distal half and third, respectively. ***Ventral vestiture.*** Setae on metanepisternum and metasternum simple, on mesanepisternum bifid. Sclerolepidia very small elongated scales.

#### Male.

Near identical to female.

#### Key

(Wood 1982). Keys to couplet 25, with no further match.

#### Etymology.

The Latin name *mundus* is a masculine adjective, meaning clean, pure, or neat, referring to the small size of the species, with few, very fine, setae on elytra and in the female frons, and the generally shiny appearance.

#### Biology and distribution.

This species is only known from the type locality at 500 m altitude in Costa Rica. Specimens were collected from three different fogging events (http://viceroy.eeb.uconn.edu/alas/canopy03.html). The species is morphologically very similar to other small *Scolytodes* species breeding in *Clusia*.

**Figures 37–45.**
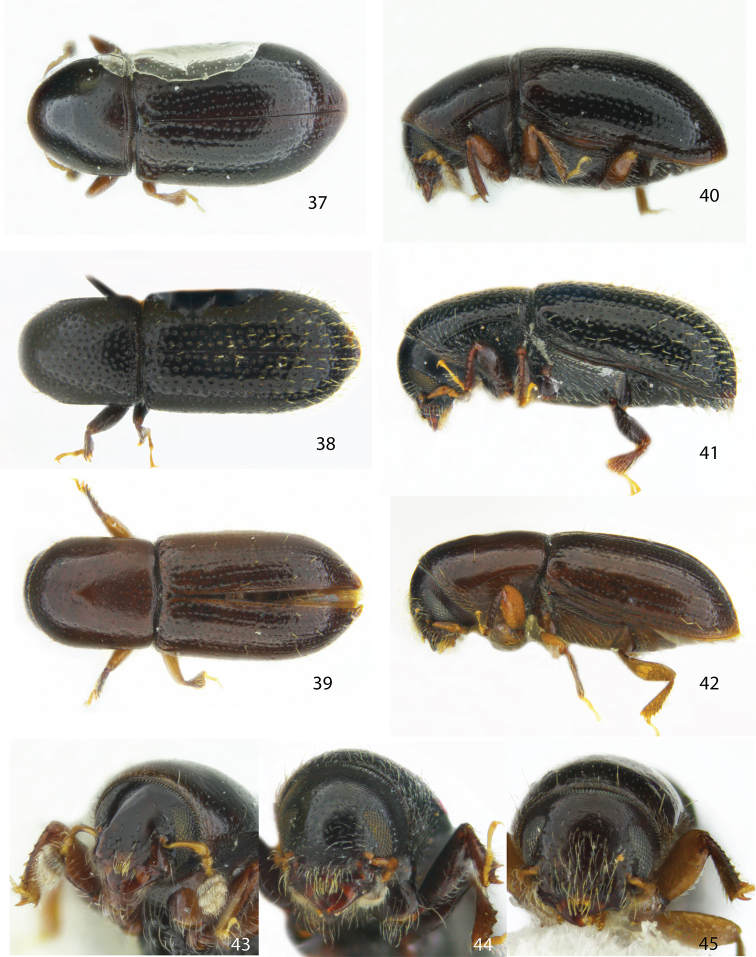
Dorsal, lateral and frontal view of the female holotype of **37, 40, 43***Scolytodesplanifrons***38, 41, 44***S.porosus***39, 42, 45***S.mundus*.

### Scolytodes callosus

Taxon classificationAnimaliaColeopteraCurculionidae

Jordal & Kirkendall sp. nov.

http://zoobank.org/1CE53A64-A4CE-4D1D-9A3E-7B03D0CD9B3E

Figs 46 , 49 , 52

#### Type material.

**Holotype, female**: Costa Rica, Prov. Heredia, 16 km SSE La Virgen, 1050–1150 m, 10°16'N, 84°05'W, 19 Feb. 2001, INBio-OET-ALAS transect, #010219-5, ex dead branch of *Clusia* [L. Kirkendall, leg]. **Allotype male**: same data as holotype. **Paratypes**: same data as holotype (2); Heredia, near Virgen de Socorro, 10°14.4'N, 84°07.5'W, 1475 m, 11 Apr. 2005, #050411-1, ex *Clusia* 8 mm dia. [L. Kirkendall, leg] (4); Prov. Heredia, 16 km SSE La Virgen, 1050–1150 m, 10°16'N, 84°05'W, 21 Mar. 2001, INBio-OET-ALAS transect, 11/TN/16/016 and 11/TN/06/011 (2); Prov. Heredia, 9 km NE Vara Blanca, 1450–1550 m, 10°14'N, 84°06'W, 6 Apr. 2005, INBio-OET-ALAS transect, #050406-3, ex dead branch of *Clusia* [L. Kirkendall, leg] (1); 17 Apr. 2005, 15/TN/08/024 (1); 20 Mar. 2005, 15/TN/11/016 (1); 20 Feb. 2001, 15/TN/04/002 (1); Prov. Heredia, 10 km SSE La Virgen, 450–550 m, 10°20'N, 84°05'W, 16 Mar. 2003, INBio-OET-ALAS transect, #030316-2 ex *Clusia* 2 cm dia. [L. Kirkendall, leg] (2); 11 km SSE La Virgen, 250–350 m, 10°21'N, 84°03'W, 11 Apr. 2004, INBio-OET-ALAS transect, #040411-1 [L. Kirkendall, leg] (1). Holotype, allotype and 8 paratypes are deposited in MNCR, 4 paratypes in ZMBN, 4 paratypes in USNM.

#### Diagnosis.

Interstriae 10 carinate to level of ventrite 1; protibiae with an additional mesal tooth near base of tooth 2. Distinguished from the very similar *S.mundus* by the impressed lower female frons which has a small median callus just above epistoma, by the sparse, but regular, interstrial punctures, by the more broadly separated pro- and mesocoxae, and by the slightly emarginated eyes which are less broadly separated above.

#### Description female.

Length 1.2–1.5 mm, 2.4–2.5 × as long as wide; color dark brown. ***Head.*** Eyes weakly emarginated along anterior margin, separated above by 2.4–2.6 × their width. Frons weakly impressed on a semi-circular area from just below upper level of eyes to near epistoma, surface strongly reticulate, with dense small punctures; a short median vertically elongated callus just above epistoma. Vestiture consisting of a mixture of fine short setae, and longer semi-erect setae, in impressed area. Antennal club with two recurved sutures on anterior face marked by short setae, on posterior side procurved, forming two oblique rings around the club; funiculus not clearly visible, likely 5-segmented. ***Pronotum*** strongly reticulate, with shallow tiny obscure punctures spaced by 3–5 × their diameter; faint asperities present on anterior fifth. Vestiture consisting of 8 erect long setae (4–2–2). ***Elytra*** generally smooth and shiny, sub-rugose on interstriae 1–3; striae 1 impressed, others not, punctures small, deep, associated with a micro-puncture and together appears like one elongated puncture, each pair separated in rows by less than their length; interstriae 3–4 × as wide as striae, with few small scattered punctures. Interstriae 10 carinate to level of ventrite 1. Vestiture consisting of about 25 erect setae regularly distributed on odd-numbered interstriae. ***Legs.*** Procoxae separated by 0.7 × and mesocoxae 1.1 × the width of one procoxa. Protibiae narrow, lateral teeth 1 and 2 of equal size, with 1–2 additional small granules along the lateral edge towards base; an additional mesal tooth present near base of tooth 2; protibial mucro obtuse. Meso- and metatibiae with 7 and 6 small socketed lateral teeth on distal half and third, respectively. ***Ventral vestiture.*** Setae on metanepisternum and mesanepisternum bifid, on metasternum and part of metanepisternum simple. Sclerolepidia large, plumose-like scales.

#### Male.

Similar to female, except frons convex, flattened just above (and level with) epistoma, with fine punctures and scant fine setae.

#### Key

(Wood 1982). Keys to couplet 25, with no further match.

#### Etymology.

The Latin name *callosus* is a masculine adjective, meaning hard, thick skin, referring to an elevated median callus just above the epistoma in females.

#### Biology and distribution.

This species is known from rainforest localities on the northern slopes of Braulio Carrillo in Costa Rica. Specimens were dissected from thin branches and twigs of *Clusia*, or collected by flight intercept traps (TN). The majority were collected in cloud forest. Some specimens were from the same twig as the larger species *S.catinus*.

### Scolytodes parvipilus

Taxon classificationAnimaliaColeopteraCurculionidae

Jordal & Kirkendall sp. nov.

http://zoobank.org/420406F4-6583-4EF9-9BAC-D065EC221143

Figs 47 , 50 , 53

#### Type material.

**Holotype, female**: Costa Rica, Prov. Heredia, 16 km SSE La Virgen, 1050–1150 m, 10°16'N, 84°05'W, 20 Feb. 2001, INBio-OET-ALAS transect, 11/TN/16/006, INB0003209608. **Allotype male**: Tapanti, Cartago, 4000 ft, IX-17-1963, S.L. Wood, ex unknown branch, #180, *Scolytodes* sp. det. S. L. Wood. **Paratype** (1 female): same data as allotype. Holotype deposited in MNCR, allotype and paratype in USNM. **Other material.** Same data as holotype, INB0003209621; only abdomen with elytra on point.

#### Diagnosis.

Interstriae 10 carinate to apex; protibiae without additional mesal tooth. The combination of few erect interstrial setae, and ground vestiture consisting of fine recumbent strial and interstrial setae, distinguish this species from several species related to *S.chapuisi* Wood, 1977 or *S.pseudopiceus* Wood, 1969, and further from *S.venustus* Wood, 1969 by the long abundant setae in the female frons.

#### Description female.

Length 1.7–1.8 mm, 2.3–2.4 × as long as wide; color light brown. ***Head.*** Eyes entire, separated above by 1.8–1.9 × their width. Frons flattened from vertex to epistoma, surface shiny, median fifth impunctate and very weakly elevated, punctured elsewhere at base of setae. Vestiture consisting of long, golden setae arising from vertex and upper lateral areas of flattened area, tips of setae reaching level of antennal insertion. Antennal club setose, two procurved sutures weakly marked by shorter setae; funiculus not clearly visible, likely 6-segmented. ***Pronotum*** strongly reticulate, with shallow, obscure punctures spaced by 1–2 × their diameter; faint asperities present on anterior third. Vestiture consisting of 8 erect long setae (4–2–2). ***Elytra*** generally smooth and shiny; striae not impressed, punctures shallow, separated in rows by their diameter, confused with interstriae on declivity; interstriae on average 2 × as wide as striae, with confused punctures slightly smaller than in striae. Interstriae 10 carinate to apex. Vestiture consisting of about 15–20 erect setae on odd-numbered interstriae, and fine recumbent ground vestiture in both striae and interstriae. ***Legs.*** Procoxae narrowly separated by 0.2 × and mesocoxae 0.7 × the width of one procoxa. Protibiae narrow, parallel-sided, lateral teeth 1 slightly as long as 2, with 3–5 additional small rugae or granules along the lateral edge towards base; protibial mucro obtuse. Meso- and metatibiae with 7 and 6 lateral, socketed, small teeth on distal half and third, respectively. ***Ventral vestiture.*** Setae on metanepisternum and metasternum simple, on mesanepisternum bifid. Sclerolepidia very small, scale-like.

#### Male.

Similar to female, except size 1.6 mm; frons more convex, with a very weakly formed carina from epistoma to near upper level of eyes, punctures obscure, surface strongly reticulate, nearly glabrous except epistoma.

#### Key

(Wood 1982). Keys to couplet 17, with no close match to *S.venustus* or *S.pseudopiceus*.

#### Etymology.

The Latin name *parvipilus* is composed by the stem of the adjective *parvus*, meaning small, the linking vowel –i, and the noun *pilus*, meaning fine hair, referring to the small curly or recumbent fine setae on the elytra, with only a few erect, longer, setae. It is invariable.

#### Biology and distribution.

This species is known from two Costa Rican cloud forest localities – the northern slopes of Braulio Carrillo, and Tapanti. Two specimens were collected in the same flight intercept trap, and two specimens were dissected from an unidentified branch.

### Scolytodes plenus

Taxon classificationAnimaliaColeopteraCurculionidae

Jordal & Kirkendall sp. nov.

http://zoobank.org/C0E3C083-60DF-4C78-AB40-48C5CBDFAE70

Figs 48 , 51 , 54

#### Type material.

**Holotype, female**: Costa Rica, Prov. Heredia, 16 km SSE La Virgen, 1050–1150 m, 10°16'N, 84°05'W, 21 Mar. 2001, INBio-OET-ALAS transect, 11/TN/18/008, INB0003210556. **Allotype, male**: Prov. Heredia, 9 km NE Vara Blanca, 1450–1550 m, 10°14'N, 84°06'W, 20 Mar. 2005, INBio-OET-ALAS transect, 15/TN/04/012, INB0003675744. **Paratypes**: same data as allotype, except 15/TN/20/020 (INB0003675955) (1); same data as allotype except 17 Apr. 2005, 15/TN/04/022 (INB0003676180) (1); Costa Rica, Puntarenas, 5 km SW Est. Biol. Las Cruces, 1425 m, 08°46'59"N, 82°59'18"W, 22 VI. 1998, R. Anderson, wet cloud forest litter, 98-108B (2); Costa Rica, S. J., Zurquí de Moravia, 1600 m, IX-1-9-1998, FIT, C.W. & L.B. O’Brien (1). Holotype and allotype deposited in MNCR, 2 paratypes in FSCA, 1 paratype in USNM, 1 paratype in CASC, 1 paratype in ZMBN.

#### Diagnosis.

Interstriae 10 carinate to level of metacoxa; protibiae without additional mesal tooth. Unique female frons with silky white, soft setae, and one of few species bearing 10 erect pronotal setae where the additional pair is close to the center of the pronotum. Only distantly related to species such as *S.banosus* (Hagedorn, 1909) and *S.hagedorni* (Schedl, 1962); more similar to *S.obovatus* Jordal, 2013 but the new species differs by having a short interstriae 10.

#### Description female.

Length 1.4–1.7 mm, 1.9–2.0 × as long as wide; color brown to dark brown. ***Head.*** Eyes weakly sinuate, separated above by 2.0–2.5 × their width. Frons flattened between eyes from vertex to epistoma, more deeply impressed at level of antennal insertion; surface shiny, smooth, median third impunctate, with dense punctures elsewhere associated with setae. Vestiture consisting of short, soft, silky setae from vertex to epistoma, except in central impunctate area. Antennal club setose, particularly on anterior face, on posterior face with two obliquely procurved sutures, segment 1 large, corneous; funiculus 6- or 7-segmented, segments very thin and disc-like. ***Pronotum*** shiny, very weakly reticulate, with shallow, medium-sized punctures spaced on average by their diameter. Vestiture consisting of 10 erect long setae (4–2–2–2), and short, fine, recumbent setae on anterior half. ***Elytra*** generally smooth and shiny; striae not impressed, punctures shallow, tiny, in longitudinal pairs, each pair separated in rows by their length; interstriae 5 × as wide as striae, punctures of same size as in striae, mainly in rows. Interstriae 10 carinate to level of metacoxa. Vestiture consisting of irregularly placed, erect, interstrial setae of variable length and thickness, and densely placed, fine, short, partly curled, semi-recumbent setae in both striae and interstriae. ***Legs.*** Procoxae separated by 0.6 × and mesocoxae 0.9 × the width of one procoxa. Protibiae narrow, oval-parallel-sided, lateral teeth 1 and 2 long and sharp, 2 as long as 1, with 3–4 additional small, sharp spines or granules along the lateral edge towards base; protibial mucro obtuse. Mesotibiae with 6–9 lateral, socketed, small teeth on distal half, the apical 3–4 teeth smaller, forming a dense comb; metatibiae with 5–6 lateral, small, socketed teeth on distal half. ***Ventral vestiture.*** Setae on metanepisternum and metasternum simple, on mesanepisternum trifid. Sclerolepidia very small, scale-like.

#### Male.

Similar to female, except frons slightly more convex, with fewer and shorter setae distributed near inner margin of eyes and antennal insertion.

#### Key

(Wood 1982). There is a mismatch in couplet 6 (short interstriae 10, but no mesal tooth on protibiae).

#### Etymology.

The Latin name *plenus* is a masculine adjective, meaning plump or chubby, referring to the stout body shape.

#### Biology and distribution.

This species is known from four Costa Rican cloud forest localities – in the northern and southern slopes of Volcan Barva (Braulio Carrillo) and close to the Panama border (Las Cruces). Two specimens were collected by leaf litter sifting, and four specimens by flight intercept trapping.

**Figures 46–54.**
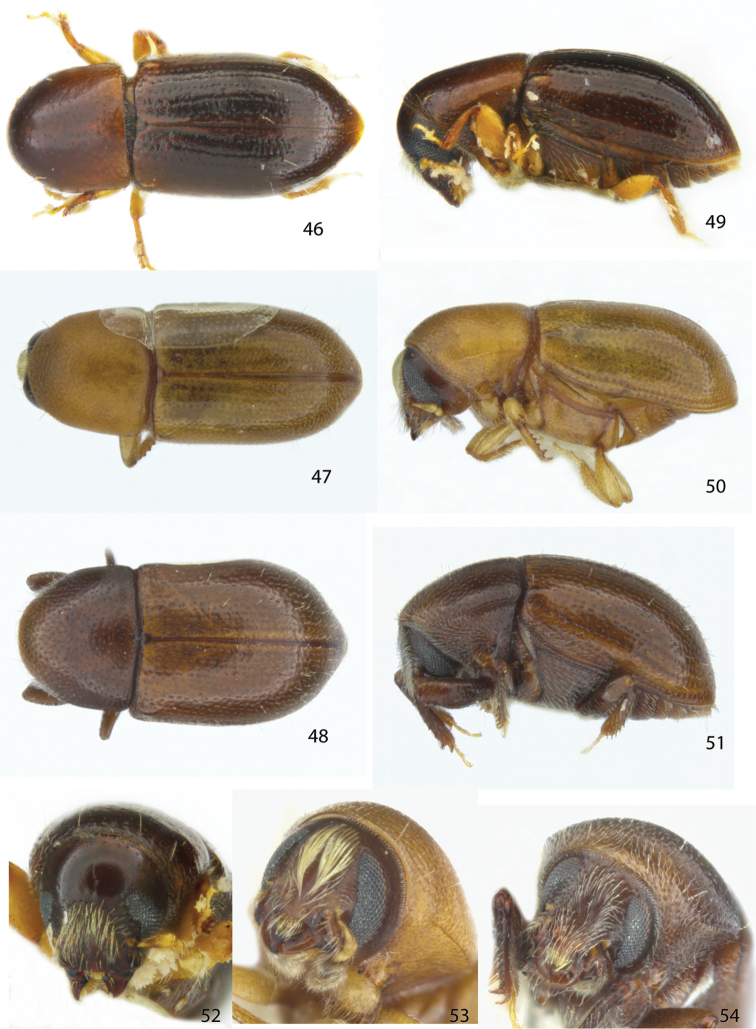
Dorsal, lateral and frontal view of the female holotype of **46, 49, 52***Scolytodescallosus***47, 50, 53***S.parvipilus***48, 51, 54***S.plenus*.

### Scolytodes minutissimus

Taxon classificationAnimaliaColeopteraCurculionidae

Schedl

Figs 55 , 56 , 57

Scolytodes minutissimus Schedl, 1952: 355.

#### Material examined.

**Holotype, female**: Costa Rica, Limón, Hamburgfarm on Reventazón. [NHMW]. **New records**: Costa Rica, Heredia, Est. Biol. La Selva, 50–150 m, 10°26'N, 84°01'W, May 2000, INBio-OET; 09 May 2000, *Goethalsiameiantha* FOT/47/29 (INBIOCRI002730681), FOT/49/26 (INBIOCRI002731590), FOT/49/23 (INBIOCRI002731568).

#### Diagnosis.

Interstriae 10 elevated to near apex; protibiae without an additional mesal tooth. Recognized by the presence of exactly two erect setae on the median anterior margin of the pronotum, and one erect setae on each interstriae 3 on the elytral disc, and by the broad longitudinal callus in a sparsely setose female frons.

#### Description female.

Length 1.3 mm, 2.0 × as long as wide; color brown. ***Head.*** Eyes entire, separated above by 1.6 × their width. Frons impressed in a hoof-shaped fashion, from broadly impressed on epistoma to more narrowly impressed near upper level of eyes, a longitudinal, broad callus in the middle of impressed area; surface shiny, punctured in impressed area only; vestiture consisting of fine, short setae in impressed area. Antennal club densely pubescent, without sutures, posterior face partly corneous on basal median half. Funiculus 5-segmented. ***Pronotum*** shiny, small punctures spaced by 3–4 × their diameter. Vestiture consisting of two erect setae at the middle of frontal margin (2–0–0). ***Elytra*** smooth, striae generally not impressed, punctures minute and barely visible, close to each other in irregular rows; interstriae approximately 4–6 × as wide as striae, punctures as small as in striae, in rows, separated by 5–10 × their diameter. Interstriae 10 carinate to near apex. Vestiture consisting of two erect setae on disc, one on each interstriae 3. ***Legs.*** Procoxae separated by 0.8 × and mesocoxae 1.0 × the width of one procoxa. Protibiae narrow, teeth 1 and 2 equal, with 3–4 additional tiny granules along the edge towards base; protibial mucro tiny, curved posteriorly. Mesotibiae with 6 socketed lateral teeth on distal half, metatibiae with 4 small socketed teeth on distal fourth. ***Ventral vestiture.*** Setae on metasternum and metanepisternum simple.

#### Male.

Similar to female except frons convex, slightly impressed just above epistoma having scant setae; surface shiny, with few punctures.

#### Biology and distribution.

This species is only known from two nearby sites in the lowland of eastern Costa Rica. Three specimens were collected by fogging of a *Goethalsiameiantha* tree (Malvaceae).

#### Note.

The holotype is a light-colored teneral specimen. The mature color is very dark brown. Wood (1982) did not note the peculiar pattern of setae with two erect frontal setae on pronotum, and two erect setae on the elytral disc (one each on interstriae 3).

**Figures 55–57.**
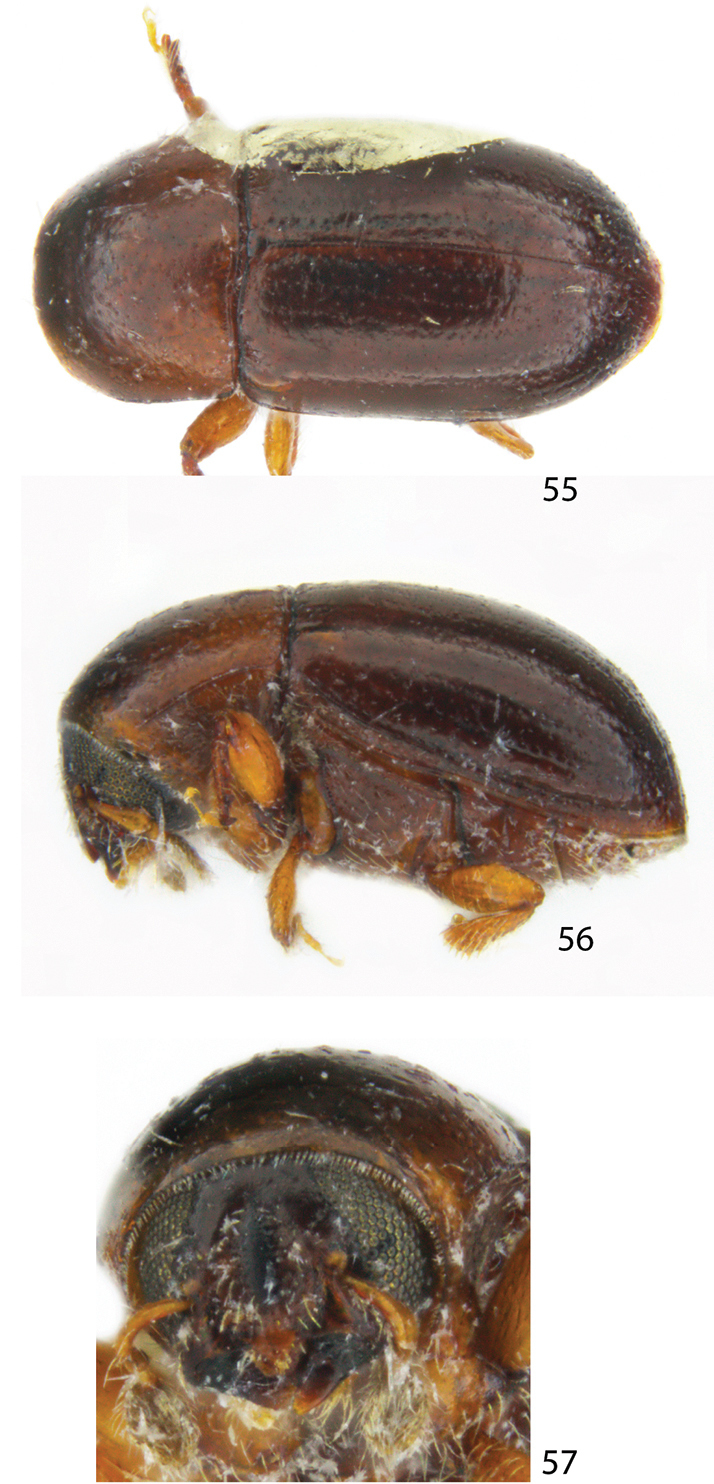
Dorsal, lateral and frontal view of a female *Scolytodesminutissimus*.

### Scolytodes costabilis

Taxon classificationAnimaliaColeopteraCurculionidae

Wood, 1974

Scolytodes obesus Wood, 1975, new synonymy

#### New Costa Rica records.

San José, Zurquí de Moravia, 1600 m, 11 July 1997, L. R. Kirkendall, 4 cm dia. *Cecropiainsignis* branch (14); Prov. Heredia, 16 km SSE La Virgen, 1050–1150 m, 10°16'N, 84°05'W, 20 Feb. 2001, INBio-OET-ALAS transect, 11/TN/19/009, INB0003210610 (1); Heredia, Est. Biol. La Selva, 50–150 m, 10°26'N, 84°01'W, 15 May 2000, FOT/50/34, fogging *Goethalsiameiantha*, INBIOCRI002731022 (1); [Puntarenas] La Gamba, Piedras Blancas NP, Esquinas rainforest, 18–28 May 2006, 8°42'/-83°12', 90–200 m, leg. Erwin Holzer (1).

*Scolytodesobesus* was described from a single teneral specimen from the Canal Zone in Panama (Wood 1975). This specimen is slightly stouter than the holotype of *S.costabilis* (2.1 vs. 2.3 × as long as wide), and the declivity of the elytra is slightly more gradual. Slight variation observed among populations and even within series of the new collections argue for there being just a single species.

*Scolytodescostabilis* is recorded for the second time from Costa Rica (see Jordal 2018), from three additional localities. With the four records reported here, this species is regarded as broadly distributed, ranging from Veracruz, Mexico (holotype of *S.costabilis*) to central Panama, from near sea level to cloud forest.

##### New country records for Hexacolini

Six species of *Scolytodes* and one of the related genus *Pycnarthrum* were recorded from Costa Rica for the first time (Table 1).

***Scolytodesclusiacolens*** Wood was previously known from Mexico and Honduras. The collection of this large species comprises three pairs just starting galleries in 8–10 mm thick *Clusia* branches.

***Scolytodescrinalis*** Wood, 1978, another *Clusia*-associated species, was previously known only from Venezuela. It was collected in two different traps.

***Scolytodeslibidus*** Wood, 1978, another Venezuelan species, was collected by traps and from leaf litter samples from several Costa Rican localities.

***Scolytodesreticulatus*** Wood, 1961 was originally recorded from *Ficus* in Mexico and the Costa Rica collection from a fallen *Ficusjimenezii* tree suggests it might be widely distributed fig tree specialist.

***Scolytodesculcitatus*** (Blandford), was known only from the holotype collected in Panama, despite being one of the three first described species in the genus (Blandford 1897). This distinctive species is unique in the genus in for having a large patch of dense long setae on the anterolateral area of the pronotum, obscuring a large impression there. The impression with long setae is likely to be a repository for fungal spores, but nothing is known of the biology of this elusive species.

***Scolytodesspadix*** (Blackman, 1943) seems to be common in Costa Rica based on the multiple collections reported here, though all are from the Caribbean side of Costa Rica; it was previously known from a single specimen taken from a mahogany log thought to have originated in Guatemala. The two host records reported here are from unrelated tree species in the families Meliaceae and Urticaceae.

***Pycnarthrumfulgidum*** Wood, 1977 was known only from the original series collected from a broken log of *Guarea* (Meliaceae) in lowland Colombia. The new record for Costa Rica is based on a male found in a Malaise trap collection in secondary forest at La Selva Biological Station.

**Table 1.** Species of *Scolytodes* not previously found in Costa Rica. Coll=collection. FIT=flight intercept trap, FOG=canopy fogging, MAL= Malaise trap, WFL=Winkler funnel litter samples. Full specimen data are given in Suppl. material 1: Table S1.

Species	Sites (specimens)	Previous range	Costa Rica province	Altitude	Host or Coll. Method
*S.clusiacolens* Wood	1 (6)	Mexico, Honduras	Heredia	1450–1550 m	*Clusia*, small branch
*S.crinalis* Wood	2 (2)	Venezuela	Heredia	1450–2050 m	FIT, MAL
*S.culcitatus* (Blandford)	1 (2)	Panama	Cartago	2800–3000 m	unknown
*S.libidus* Wood	4 (5)	Venezuela	Cartago, Heredia	1950–2600 m	FIT, MAL, WFL
*S.reticulatus* (Wood)	1 (3)	Mexico	San José	1200 m	*Ficus* branch
*S.spadix* (Blandford)	7 (10)	Guatemala? (see text)	Heredia	50–550 m	*Carapa*, *Coussapoa*, FOG, MAL

## Supplementary Material

XML Treatment for Scolytodes

XML Treatment for Scolytodes angulus

XML Treatment for Scolytodes niger

XML Treatment for Scolytodes simplex

XML Treatment for Scolytodes sufflatus

XML Treatment for Scolytodes squamatifrons

XML Treatment for Scolytodes comosus

XML Treatment for Scolytodes spatulatus

XML Treatment for Scolytodes seriatus

XML Treatment for Scolytodes profundus

XML Treatment for Scolytodes catinus

XML Treatment for Scolytodes fimbriatus

XML Treatment for Scolytodes sulcifrons

XML Treatment for Scolytodes planifrons

XML Treatment for Scolytodes porosus

XML Treatment for Scolytodes mundus

XML Treatment for Scolytodes callosus

XML Treatment for Scolytodes parvipilus

XML Treatment for Scolytodes plenus

XML Treatment for Scolytodes minutissimus

XML Treatment for Scolytodes costabilis

## Supplementary materials

Supplementary material 1 Table S1 Data type: occurrences Explanation note: New records of Scolytodes and Pycnarthrum from Costa Rica. Species marked in bold are new country records, see Table 1 in text. This dataset is made available under the Open Database License (http://opendatacommons.org/licenses/odbl/1.0/). The Open Database License (ODbL) is a license agreement intended to allow users to freely share, modify, and use this Dataset while maintaining this same freedom for others, provided that the original source and author(s) are credited.Bjarte H. Jordal, Lawrence R. Kirkendall
